# Supplementary figures and images for: cis-regulatory architecture of a short-range EGFR organizing center in the Drosophila melanogaster leg
Source: PLoS Genet. 2018 Aug 24;14(8):e1007568. doi: 10.1371/journal.pgen.1007568 (PMC6147608; doi:10.1371/journal.pgen.1007568)

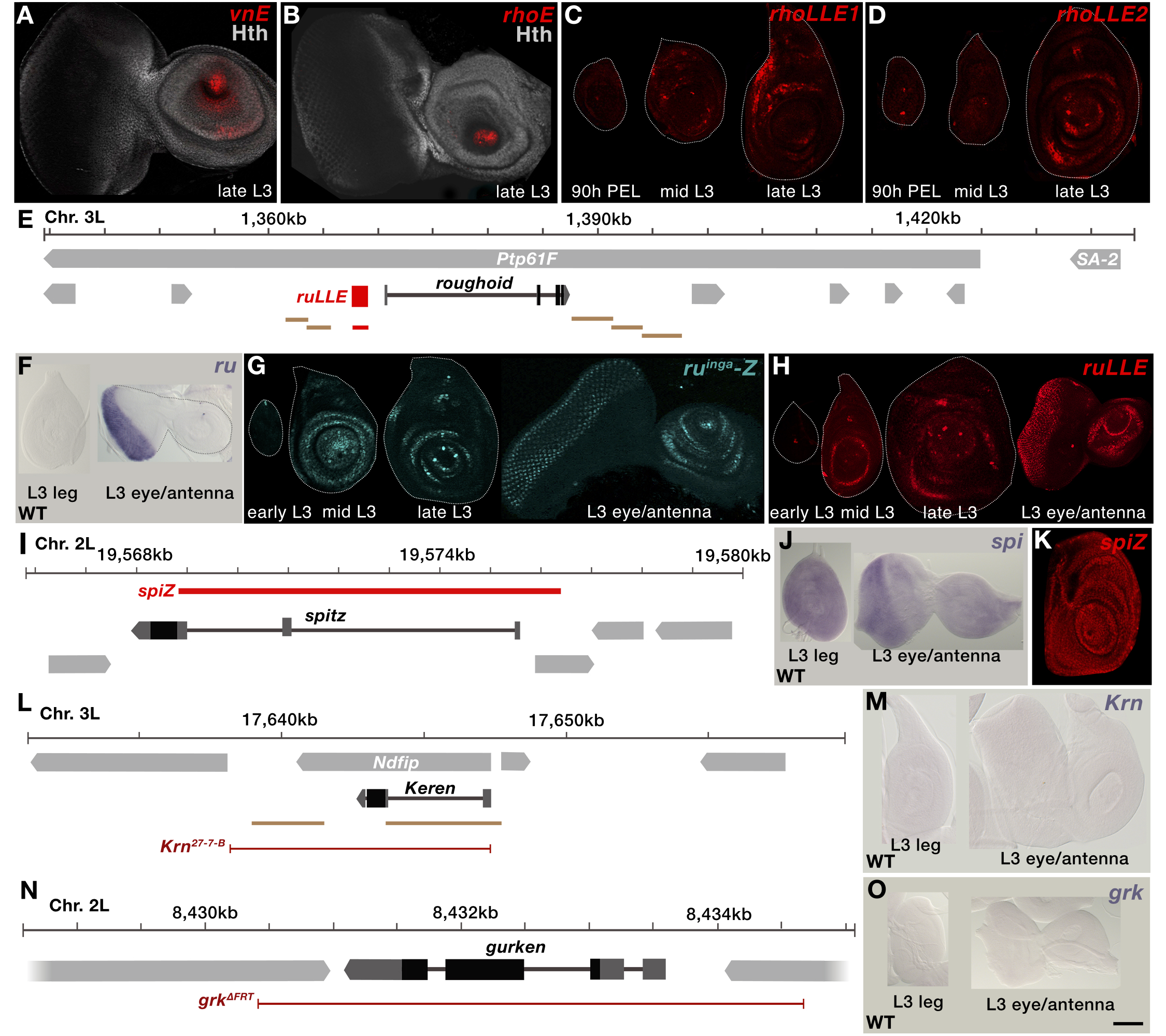

Supplement: S1 Fig — (A-B) Expression pattern of vnE (A) and rhoE (B) in third instar eye-antennae discs. (C-D) Expression pattern of (C) rhoLLE1 and (D) rhoLLE2 throughout leg disc development. (E) Schematic representation of ru genomic locus with enhancer bashing results. Fragments represented in tan did not drive expression in leg discs. (F-H) Expression pattern of ru from in situ (F), ruinga (G) and ruLLE (H). (I) Schematic representation of spi genomic locus with enhancer bashing results. (J-K) Expression pattern of spi from in situ (J) and spi-lacZ reporter construct (K). (L) Schematic representation of Krn genomic locus with enhancer bashing results and Krn27-7-B mutant. Fragments represented in tan did not drive expression in leg discs. (M) Expression pattern of Krn from in situ. (N) Schematic representation of grk genomic locus with grkΔFRT mutant. (O) Expression pattern of grk from in situ. (TIF) [file pgen.1007568.s001.tif]

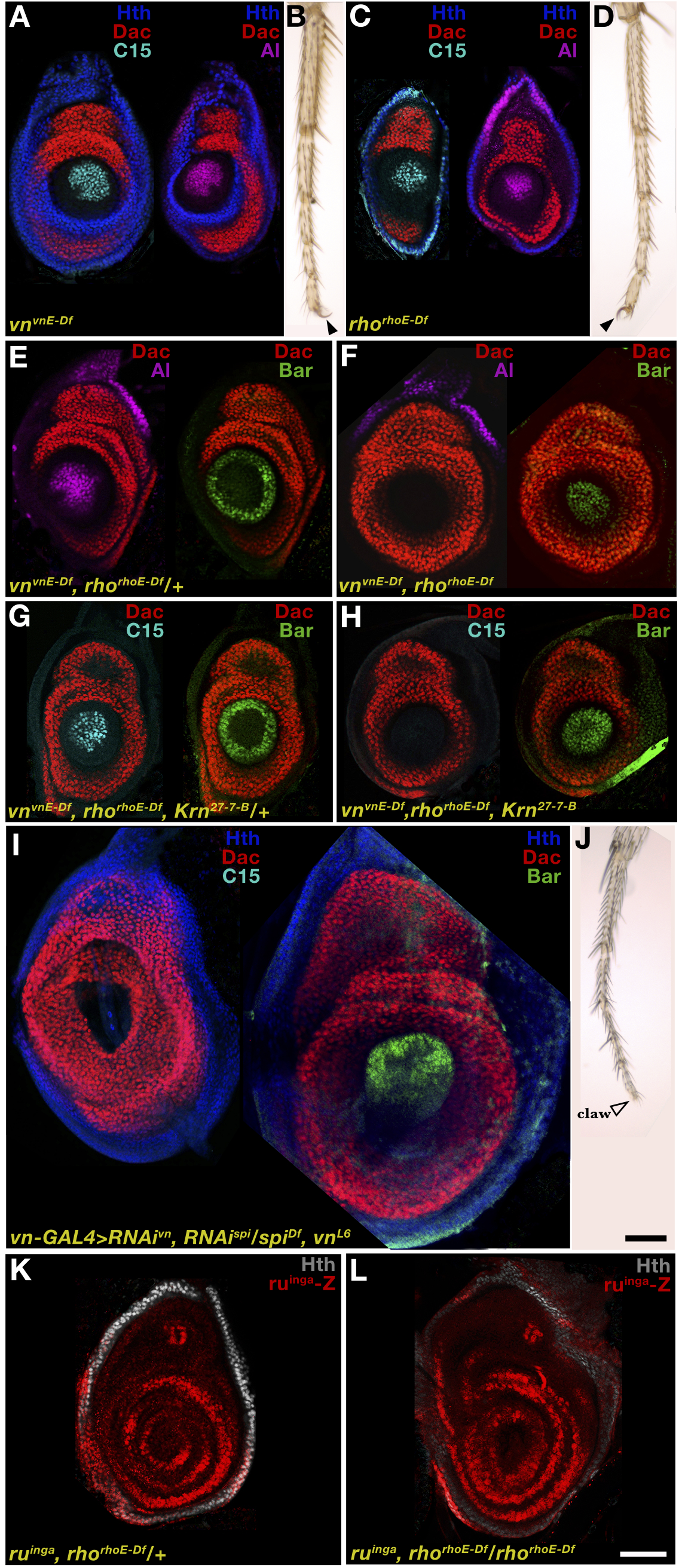

Supplement: S2 Fig — (A) Expression pattern of C15 and Al in vnvnE-Df mutant. (B) Adult leg of vnvnE-Df mutant. Filled arrowhead indicates intact pretarsal claw. (C) Expression pattern of C15 and Al in rhorhoE-Df mutant. (D) Adult leg of rhorhoE-Df mutant. Filled arrowhead indicates intact pretarsal claw. (E) Expression pattern of Al and BarH1 in WT (rhorhoE-Df vnvnE-Df/+) and (F) rhorhoE-Df vnvnE-Df double mutant. (G) Expression pattern of C15/Bar/Dac in WT (rhorhoE-Df vnvnE-Df Krn27-7-B/+) and (H) rhorhoE-Df vnvnE-Df Krn27-7-B triple mutants. (I-J) spi vn double RNAi driven by vn-GAL4. Expression pattern of C15 and BarH1 in third instar leg discs (I) and adult leg (J). Open arrowhead indicates absent pretarsal claw). (K-L) Expression pattern of ruinga-lacZ in ruinga rhorhoE-Df/+ (K) and ruinga rhorhoE-Df/ rhorhoE-Df (L) leg imaginal discs. (TIF) [file pgen.1007568.s002.tif]

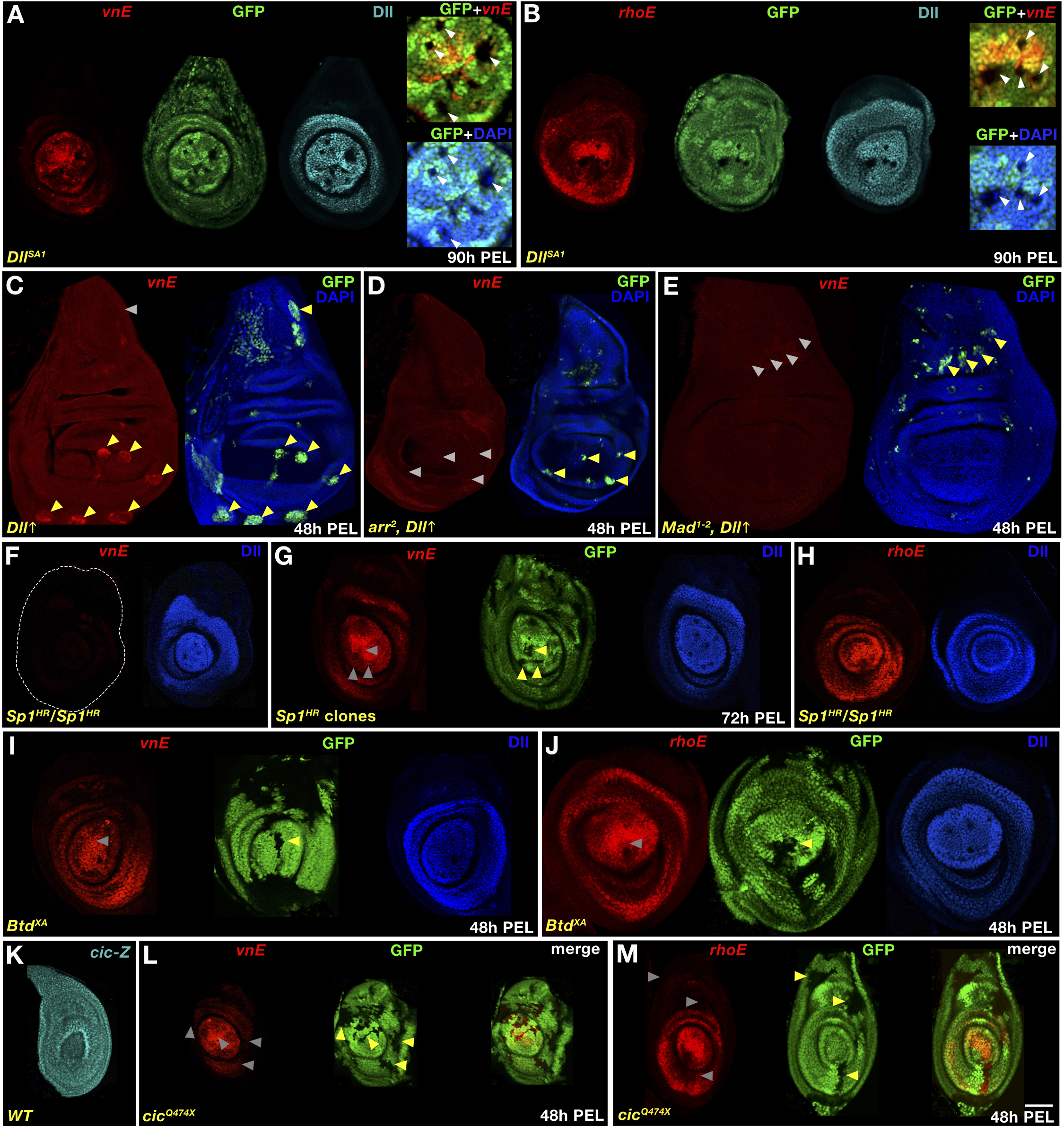

Supplement: S3 Fig — (A) vnE- or (B) rhoE-driven lacZ expression in DllSA1 mutant clones generated at 90h PEL. (C-E) vnE-driven lacZ expression in wing discs in Dll ectopic overexpression clones in WT background (C); Dll ectopic overexpression clones in arr2 mutant background (D); Dll overexpression clones in Mad1-2 mutant background (E); (C-E) clones were generated at 48h PEL. (F-G) vnE-driven lacZ expression in leg discs of Sp1HR mutant animals (F); Sp1HR mutant clones generated at 72h PEL (G). (H) rhoE-driven lacZ expression in leg discs of Sp1HR mutant animals. (I-J) vnE- (I) or rhoE- (J) driven lacZ expression in btdXA mutant clones generated at 48h PEL. (K) cic-lacZ expression in leg discs. (L-M) vnE- (L) or rhoE- (M) driven lacZ expression in leg discs with cicQ474X mutant clones generated at 48h PEL. (TIF) [file pgen.1007568.s003.tif]

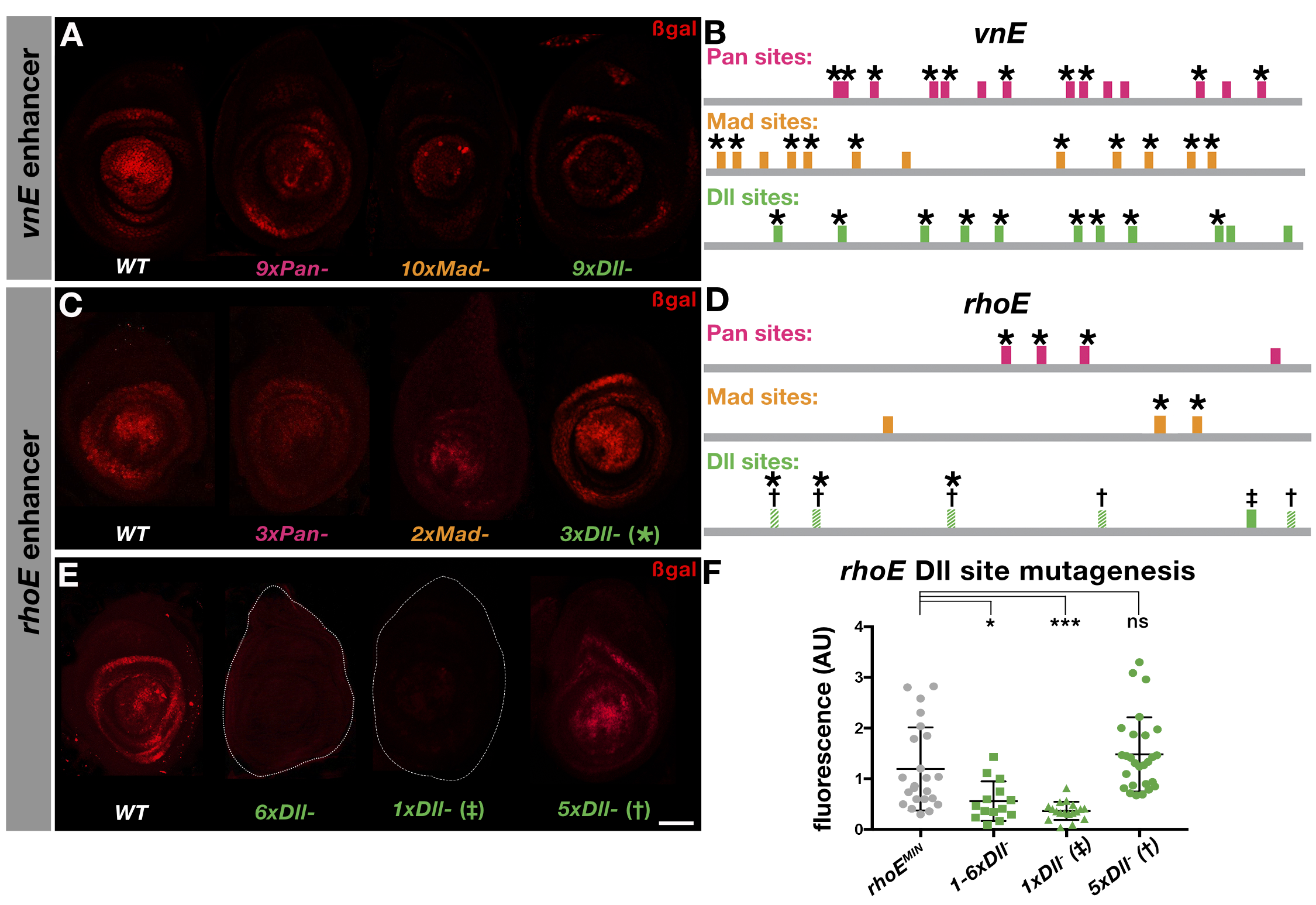

Supplement: S4 Fig — (A, C, E) vnE- (A) and rhoE- (C, E) driven expression of WT and intermediately mutant CRMs. (B and D) schematic representation of binding sites in vnE and rhoE, respectively. Mutated sites for the CRM-reporter genes shown in A, C, and E are indicated by the *, † and ‡. (F) Quantification of expression levels; fluorescence was calculated as a ratio of β-gal:Dll intensity in the center of the discs (see Methods for details). WT rhoEMIN n = 23, 6xDll n = 14, 1xDll n = 18, 5xDll n = 27 where n indicates number of leg discs analyzed. (TIF) [file pgen.1007568.s004.tif]

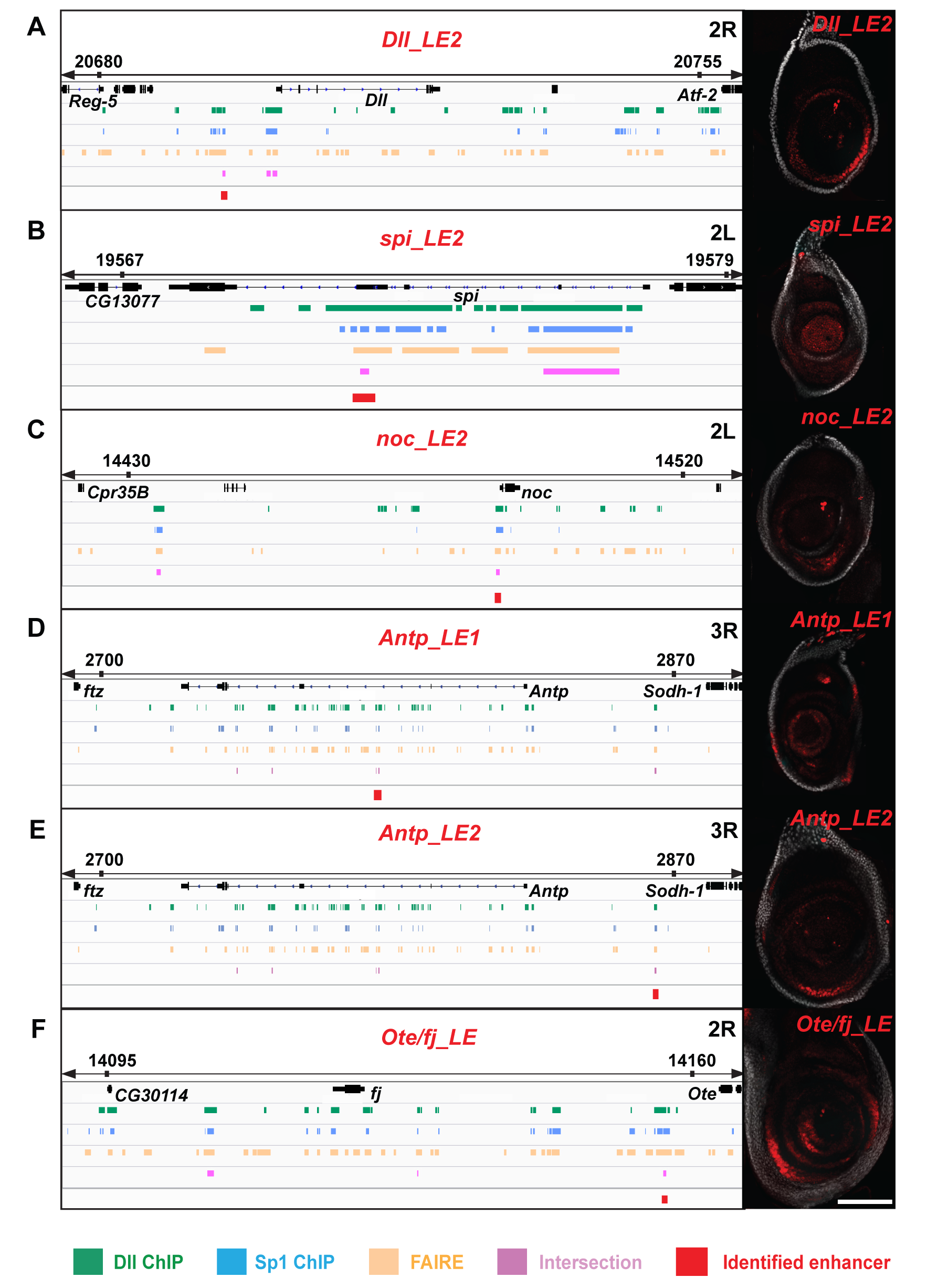

Supplement: S5 Fig — Schematic representation of identified genomic loci and the expression patterns they drive in reporter genes from Dll_LE2 (A); spi_LE2 (B); noc_LE2 (C); Antp_LE1 (D); Antp_LE2 (E); Ote/fj_LE (F). (TIF) [file pgen.1007568.s005.tif]
